# Commentary: An Initial Passive Phase That Limits the Time to Recover and Emphasizes the Role of Proprioceptive Information

**DOI:** 10.3389/fneur.2019.00404

**Published:** 2019-04-24

**Authors:** John H. J. Allum, Flurin Honegger

**Affiliations:** Division of Audiology and Neurootology, Department of ORL, University of Basel Hospital, Basel, Switzerland

**Keywords:** balance corrections, vestibulospinal signals, proprioceptive signals, vestibular loss, support surface perturbations

A recent article by Le Goic et al. (1) raises the very same issues concerning the participation of vestibular-spinal reflexes in balance corrections to support surface movement that existed in the 90's—see Peterson, 1989 (2). Then, Nashner et al. assumed (3, 4), as have now Le Goic et al. (1), that the apparently delayed onset of head movement following a support surface translations of 35 cm/s or greater suggested little or no direct vestibular contribution to balance corrections.

In order to establish a vestibular contribution, three conditions need to be fulfilled. Firstly, head angular and/or linear accelerations registered by the semi-circular canal and otolith sensory system, respectively, need to be early enough to contribute to balance corrections observed some 120 ms after the onset of the support surface perturbation (5–7). Secondly, the recorded head accelerations need to be supra-threshold for the vestibular sensory systems. Thirdly, a change in the amplitude and/or latency of muscle responses following vestibular loss needs to be established (5, 6, 8).

By comparing changes in muscle response amplitudes to translations and rotations of the support surface for bilateral lower leg proprioceptive loss patients (9), a bilateral total leg proprioceptive loss patient (10), and bilateral vestibular loss patients (8, 11), we came to the conclusion that balance corrections are normally triggered by ankle proprioceptive inputs and once triggered are modulated by both proprioceptive and vestibular inputs (9). We found no evidence that vestibular inputs trigger balance corrections (8, 9). Rather, in the absence of ankle proprioceptive inputs, those from the knee and hip trigger balance corrections (10). Once triggered, the vestibular-spinal modulation is profound if it is assumed that the difference between response amplitudes of bilateral vestibular loss patients and age-matched controls is equal to this modulation. On this basis, the activity of tibialis anterior, quadriceps, hamstrings, and abdominal muscles is enhanced and for paraspinal muscles inhibited by vestibular inputs, following a rearward support surface translation with eyes open or closed (8, 12)—see also Figures 1A,B,E,F. For a toe-up rotation of the support surface with the same amount of ankle dorsiflexion as with translation (4 deg) the vestibular modulation is different (see Figure 1), possibly because of differences in the amount and direction of initial head linear and angular accelerations (see Figures 1C,D).

**Figure 1:**
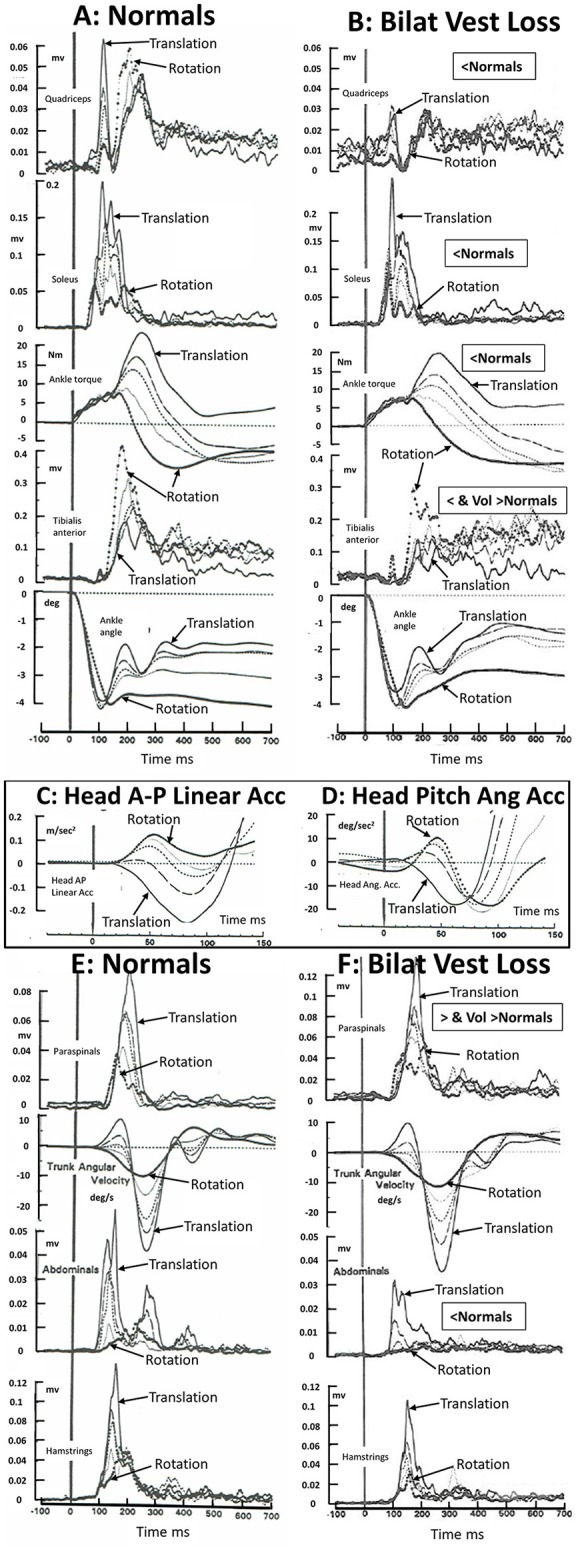
Muscle activation patterns in 16 healthy normal subjects **(A–E)** and 5 bilateral peripheral vestibular loss (BVL) subjects) **(B,F)** to 36°/s toe up rotations of the support surface, 35 cm/s backwards translation of the support surface, and 3 combinations of rotation and translation. The support surface movements were designed to elicit 4° of ankle dorsiflexion at 100 ms for all stimulus combinations. The five combinations of rotation and translation used were 4.4 deg and 0 cm (responses marked rotation in the figure); 3.5 deg and 0.7 cm; 2.8 deg and 1.4 cm; 2 deg and, 2 cm; and 0.7 deg and 3.5 cm (marked translation). The recordings were acquired under eyes open conditions. BVL areas of muscle activity and ankle torque amplitudes which were less than those of healthy controls between 80 and 240 ms post stimulus onset is labeled “<Normal” or, when activity is greater than controls in paraspinal muscles “>Normal.” Note that the scale factor for paraspinals has been reduced in F. Late onset (voluntary) muscle activity (over 250–500 ms post onset) greater than normal in tibialis anterior and paraspinals is labeled “Vol>Normal.” Head anterior-posterior (AP) linear and pitch angular acceleration recorded in the normal subjects over the first 150 ms following stimulus onset, is displayed as an insert panel, **(C,D)**, respectively. AP rearward and pitch neck flexion acceleration is in the negative direction. Note the early, 20 ms, onset of head linear acceleration **(C)** and pitch angular acceleration **(D)** with respect to ankle angle rotation (lower traces in **A,B**). All traces are average population traces, each subject's response being the average of 9 responses to the same translation-rotation stimulus combination. The combinations were presented in random order and the first response was not used for analysis to avoid startle effects entering the data analysis. Note that all muscles recorded except soleus showed an influence of BVL on muscle responses. Soleus showed a trend for an influence confirmed in other studies performed under eyes closed conditions (6, 11). The original purpose of the recordings was to confirm that changes in movement strategies with different combinations of support surface rotation or translation were not dependent on ankle proprioceptive or vestibular inputs but rather on hip proprioceptive inputs. [Data reproduced with permission from Allum et al. (8, 13)].

The crucial aspect of the above argument for a role for vestibular contributions to balance corrections is the presence of early stimulus evoked head accelerations. Considering only the pitch plane, three types of accelerations are observed; vertical and anterior-posterior linear accelerations and pitch angular accelerations (5, 7, 8, 14). For example, a toe-up rotation of the support surface with a velocity 35°/s or greater accelerates the head up and forwards, a toe-down rotation drives the head down and rearwards (7, 8); backward translation (35 cm/s) drives the head down and rearwards (7, 8, 14), and a forward translation drives the head down and forwards (14). The onsets of the AP linear accelerations are some 20 ms after the onset of ankle angular velocity following a 35 cm/s rearward translation of the support surface (7, 13, 14) and have amplitudes of 0.3 m/s^2^ (8, 13, 14) which are supra-threshold for the vestibular system (15, 16)—see Figures 1C,D. It is noteworthy that Allum et al. (8, 13) and 5 years later Runge et al. (14) observed identical latencies, 20 ms, and similar amplitudes (0.3 m/s^2^) of head AP linear acceleration for 35 cm/s rearward support surface translations despite differences in measurement techniques.

Le Goic et al. (1) did not observe these early head accelerations for support surface translations of 35 cm/s [used by Allum et al. (8) and Runge et al. (14)] and higher velocities. Differences in recording techniques may provide the reason why early head accelerations were not observed. Direct recordings of linear accelerations with accelerometers having a bandwidth of 30 Hz are capable of measuring, for example, the initial 50 ms duration pulses of initial head AP and vertical linear acceleration when sampled at 1 kHz [see Figures 1C,D and Figure 1 Allum et al. (7)]. However, one could well imagine that using position information sampled at 200 Hz, then filtering these recordings with a filter having a bandwidth of <10 Hz, would leave considerably reduced recorded amplitudes of signals at 10 Hz from which to derive low-noise head acceleration information. Indeed the conclusion would probably be reached that no early head movement had occurred (1). Regardless of how the head accelerations are measured, the most logical conclusion concerning the differences between amplitude of balance correcting muscle responses to support surface perturbations following bilateral peripheral vestibular loss (5, 6, 8, 11) when compared to those of healthy controls (see Figure 1) is that the lack of responses of the vestibular sensory system in the vestibular loss subjects to supra-threshold head accelerations (for healthy controls) must underlie the difference in response modulation seen in the 6 muscles shown in Figure 1. Interestingly, the same argument about the lack of head movement indicating no direct vestibular involvement in balance corrections was initially made by Nashner et al., albeit based on a motion analysis system with a sampling rate of 10 Hz (3), so that head acceleration pulses of 50 ms would have been occurring between the 100 ms sample intervals.

Hopefully, these measurement issues can be resolved in future studies employing wearable micro-electro-mechanical motion sensors to measure head accelerations. Then, whether or not early head accelerations, which could elicit vestibular-spinal reflexes, are observed for perturbations to stance and gait (17) should be clearer.

## Author Contributions

JA wrote the first draft and contributed to data collection. FH revised the manuscript and contributed to data collection.

### Conflict of Interest Statement

The authors declare that the research was conducted in the absence of any commercial or financial relationships that could be construed as a potential conflict of interest.
